# The health of women in the US fire service

**DOI:** 10.1186/1472-6874-12-39

**Published:** 2012-10-31

**Authors:** Sara A Jahnke, WS Carlos Poston, C Keith Haddock, Nattinee Jitnarin, Melissa L Hyder, Cheryl Horvath

**Affiliations:** 1 Center for Fire Rescue & EMS Health Research, Institute for Biobehavioral Health Research, National Development & Research Institutes, 1920 W 143rd St, Ste 120, Leawood, KS, 66224, 913-681-0300, USA; 2 Northwest Fire District, 5225 W Massingale, Tucson, AZ, 85743, USA

**Keywords:** Fire fighters, Women, Occupational health, Alcohol, Tobacco, Body composition

## Abstract

**Background:**

Despite statements from national fire service organizations, including the International Association of Fire Fighters (IAFF) and the International Association of Fire Chiefs (IAFC), promoting a diverse work force related to gender within the fire service, rates of women firefighters remain very low. Thus, research into why this extensive gender disparity continues is a high priority. Recent years have seen a number of large scale studies on firefighter health and health risk behaviors however, none have focused on the health of women firefighters and nearly all have eliminated women from the sample due to small sample size. Data from the present report is drawn from all females in a large, randomly selected cohort of firefighters in an epidemiological study designed to assess health outcomes and health risk behaviors identified as most important to the fire service.

**Methods:**

Data reported for the present study were collected as baseline data for the Firefighter Injury and Risk Evaluation (FIRE) Study, a longitudinal cohort study examining risk factors for injury in both career and volunteer firefighters in the IAFC Missouri Valley Region. Of the departments assessed, only 8 career and 6 volunteer departments had any women firefighters. All the women solicited for participation chose to enroll in the study. The number of women ranged from 1 to 7 in career departments and 1 to 6 in volunteer departments.

**Results:**

Where possible, comparisons are made between female firefighters and published data on male firefighters as well as comparisons between female firefighters and military members. Compared to male firefighters, females had more favorable body composition among both career and volunteer firefighters. Tobacco use rates were generally higher among females than males and rates among female firefighters were similar to the rates of female military members. While rates of alcohol use were higher than the general population, only one of the participants evidenced responses in the range of concern on the CAGE screening.

**Conclusions:**

In general, the findings offer an interesting glimpse of the health of women in the fire service as a generally healthy occupational workforce with some unique health risk behavior challenges. They also highlight some of the similarities and differences between male and female firefighters and bolster the argument for studying female firefighters as a unique occupational sub-population.

## Background

According to the *National Report Card on Women in Firefighting*[1], of the over 300,000 paid firefighting personnel in the country, only an estimated 3.7% are women. The Bureau of Labor Statistics estimates the number at 5.1% [2]. In the US, there are 291 departments considered “metropolitan” (i.e., more than 400 paid personnel) and more than half of these departments (51.2%) have no women firefighters [1]. The largest fire department in the country, the New York City Fire Department, is comprised of less than a quarter of one percent (.0025) women. Although women are 50.9% of the workforce, their representation in the fire service is uniquely low, even when compared to occupations with similar demands and traditions [1]. Hulett and colleagues^1^ examined the proportion of women in traditionally male-dominated occupations with similar job requirements with regard to strength, stamina, and danger (e.g., loggers, roofers, septic tank servicers) and found that women constitute 17% of these occupations. Rates of women in the police force are estimated to be around 13% [2] and approximately 14% of active duty military personnel are female [3]. Even in the United States (US) Marine Corps, where all positions require infantry expertise, the rate of female personnel is higher (6%) than the fire service [3].

A target goal of 16-22% women in the fire service was established as legally defensible by a recent court decision [4]. Unfortunately, there are no data to suggest that the proportion of women in the fire service is increasing. According to *iWomen,* the largest organization of female firefighters in the US, the low proportion of female firefighters results from a combination of limited recruitment and poor retention [5]. Despite statements from national fire service organizations, including the International Association of Fire Fighters (IAFF) [6] and the International Association of Fire Chiefs (IAFC) [7], promoting a diverse work force related to gender within the fire service, rates of women firefighters remain very low. Thus, research into why this extensive gender disparity continues is a high priority.

A number of explanations for the very low proportion of female firefighters have been posited including the psychological and physical strain of firefighting, gender-based harassment, and a culture that is generally not accepting of women [1]. While worksite harassment encountered by women firefighters has been widely recognized and studied as an impediment to recruitment and retention [1,8], relatively limited attention has been paid to the health and safety implications of the profession for women. More than half (58%) of women surveyed by iWomen reported ill-fitting protective gear [4] including 14% of women who reported ill-fitting self-contained breathing apparatus (SCBA) face pieces, critical for protecting firefighters against toxic air particles. Many women firefighters reported that they were unable to achieve an adequate seal, thereby dramatically increasing exposure to harmful chemicals at high concentrations.

Concerns also exist about the negative impact of occupational exposure on reproductive health [9]. Research conducted in the 1980’s suggested that toxic exposures experienced by male firefighters may increase the likelihood of birth defects among their offspring [10]. Similarly, preliminary data from the same period indicated that the exposure to carbon monoxide and high ambient temperatures may increase the risk of birth defects among pregnant female firefighters and highlight the reproductive dangers of firefighting [11,12]. Evidence also suggests that pregnant women’s exposure to loud noises (e.g. air horns, sirens) may lead to lower fetal weight and increased risk of fetal mortality [10]. Despite calls over the past decade for additional research into the impact of occupational exposures on the reproductive health of female firefighters [13] and preliminary evidence that exposures may negatively impact reproductive health, there remains almost no scientific literature on the reproductive health of female firefighters.

A growing body of literature highlights the uniquely high risk firefighters face from a large number of occupationally-related diseases and injury. For example, a meta-analysis of both male and female firefighters by LeMasters and colleagues [14] indicated increased cancer risk due to exposures faced by firefighters. An assessment of the leading causes of line of duty deaths (LODD) from 1994 to 2004 among firefighters found that more firefighters died from cardiovascular disease (CVD)-related events than any other cause on the fire ground [15]. Goebe et al. [16] examined risk of death during various firefighting duties among male firefighters and found that firefighters were substantially more likely to die from CVD-related events during: 1) fire suppression activities; 2) the time period of alarm response; and 3) alarm returns when compared to nonemergency tasks.

Preliminary evidence also suggests that rates of overweight and obesity (BMI ≥ 25) are higher in the fire service than the general public. For instance, Soteriades [17] found that 77% of male firefighters studied were overweight or obese. Subsequent analyses indicated that higher BMI was related to higher blood pressure, worse metabolic profiles, and lower exercise tolerance. In the first population-based cohort study of firefighter cardiovascular risk factors conducted to date, our group found 79.5% rate of overweight and obesity and among male firefighters that obese firefighters evidenced significantly lower physical fitness than their normal weight peers [18]. Finally, likely due to unhealthy body composition and poor fitness along with strenuous work demands, firefighters are at high risk of musculoskeletal injuries. The financial cost of injuries among firefighters is estimated to be between $2.7 and $7.8 billion dollars per year [19]. Despite the increased attention to firefighter health and wellness, there remains almost no focus on how the demands of the occupation uniquely affect women. In fact, studies of firefighter health often do not mention gender or exclude females from the analyses [16,18,20].

In the mid-1990s, the Federal Emergency Management Agency (FEMA) and the US Fire Administration (USFA) convened a panel to discuss the health and safety issues of female emergency responders [21]. While the knowledge gap related to female firefighters was clearly identified and general domains for research have been enumerated, over a decade later there remains no published research on any of the domains identified as important to women in the fire service. A report published by the National Fallen Firefighters of the 2^nd^ Fire Service Research Agenda Setting Symposium identified the lack of research on women firefighters as a particular concern and made a specific recommendation for increased research on this population. Recent years have seen a number of large scale studies on firefighter health and health risk behaviors [16-18,20,22-28] however, most do not focus on the health of women firefighters and a number have eliminated women from the sample due to small sample size. In our experience, when we have attempted to publish research including women firefighters, editors or reviewers have suggested we remove them due to the small sample size. Data from the present report is drawn from all females in a large, randomly selected cohort of firefighters in an epidemiological study designed to assess health outcomes and health risk behaviors identified as most important to the fire service in overall.

## Methods

Data reported for the present study were collected as baseline data for the Firefighter Injury and Risk Evaluation (FIRE) Study, a longitudinal cohort study examining risk factors for injury in both career and volunteer firefighters (“A prospective evaluation of health behavior risk for injury among firefighters—the Firefighter Injury Risk Evaluation [FIRE] study”; EMW-2007-FP-02571) in the IAFC’s Missouri Valley Region (Colorado, Iowa, Kansas, Missouri, North Dakota, Nebraska, South Dakota, and Wyoming). The project was granted Institutional Review Board (IRB) approval from both National Development & Research Institutes and the compliance office of FEMA.

Sampling for the present study was a one stage cluster sampling design followed by census sampling of the department personnel. Department selection was obtained using a computerized random selection program and the National Fire Department Census Database. A detailed accounting of the procedures for department solicitation can be found in Poston et al. [22].

Once approval was provided by the department, the research team traveled to each of the departments and met with crews of firefighters on all shifts while the firefighters were on-duty. The project was explained and consent was obtained from those interested in participating. Of the firefighters who listened to the explanation, 96.6% agreed to participate in the research.

Of the 24 departments assessed, 8 career and 6 volunteer departments had any women firefighters. All the women solicited for participation chose to enroll in the study. The number of women ranged from 1 to 7 in career departments and 1 to 6 in volunteer departments. In total, four women were excluded from analysis (3 career, 2 volunteer) because they held either administrative or recruit positions and did not classify themselves as active in the firefighter and/or paramedic role.

## Measures

### General health assessment

The general health question (“In general would you say your health is…” with response options from poor to excellent) from the SF12v2 Health Survey [28] was administered to address general self perceived health.

### Overweight/Obesity

Measured height and weight was used to calculate Body Mass Index (BMI;kg/m^2^). Participants will be categorized as underweight (< 18.5 kg/m^2^), healthy weight (range, 18.5–24.9 kg/m^2^), overweight (range, 25.0–29.9 kg/m^2^), or obese (≥ 30.0 kg/m^2^) [29]. Waist circumference was assessed with a spring-loaded non-stretchable tape measure based on procedures recommended by the US obesity guidelines with a measurement over 35 inches or more being considered at increased risk/obese for women [30,31]. Height was assessed by use of a portable stadiometer. Percent body fat and bodyweight were determined using the Tanita 300 bioelectrical impedance scale (Tanita Corporation of America, Inc. Arlington Heights, IL). The Tanita 300 is commonly used because of its portability and accuracy in determining percent body fat [32]. Categorization was based on US obesity guidelines with 30% or more body fat being considered obese, 25-31% acceptable, 21-24% fitness, and 14-20% athletic for women [30,31].

### Blood pressure & pulse

An Omron HEM-711AC, which digitally measures both blood pressure and pulse rate, was used for assessments. Blood pressure was assessed according to the standardized method used by the Hypertension Detection and Follow-Up Program following the standard epidemiological protocol, i.e., 5 min of rest in a seated position and then three separate blood pressure measurements [33]. Those with a systolic blood pressure between 120 and 139 mmHg were considered prehypertensive, 140 or more mmHg were classified as hypertension. For diastolic blood pressure, a reading between 80 and 89 mmHg was classified as prehypertension, a reading above 90 mmHg was considered hypertension.

### Strength & flexibility

The Jackson Strength Evaluation System, commonly used for the lift tasks of the National Institute of Occupational Safety, was used to assess isometric strength. NFPA 1500 [34] recommends the Jackson Strength Evaluation System as a possible tool for physical fitness assessment in the fire service. Also as suggested by NFPA 1500 [34], the adjustable sit and reach flexibility tester was used to assess flexibility. Both have national norms based on gender that indicate levels of performance.

### Estimated maximal oxygen consumption

The Self Report of Physical Activity (SRPA) measure was used to assess general ratings of physical activity over the past 30 days. The SRPA has been found to be valid among adult non-firefighter populations and exhibits a high correlation with measured maximal oxygen consumption (VO_2max_) [35]. For the SRPA, participants selected a value from the questionnaire that most closely indicated their physical activity over the past 30 days. The score on the questionnaire was then combined with BMI, age and gender to estimate VO_2max_ which can be classified by METS. This method has been found to have equal or better accuracy at estimating aerobic capacity then submaximal heart rate tests [36-40]. The aerobic capacity threshold of ≥ 12 METS (VO_2max_ ≥ 42 mL/kg/min) was used based on the NFPA recommended minimum post cardiac event exercise tolerance threshold which also has been found to be the necessary capacity for firefighting duties [41-43].

### Injury

Survey questions about injuries were assessed using a tool developed by our research team that combines questions from the National Health Interview Survey [44] and fire service-specific questions of injury as outlined by the National Institute of Standards and Technology [45]. This survey assessed both injury and days out due to injury (i.e., injury days, workers compensation claims data, other insurance information).

### Substance use

Tobacco and alcohol use questions were adapted from the Department of Defense Survey of Health Related Behaviors Among Personnel, the central health surveillance project for the military [46]. Participants were asked if they had ever smoked cigarettes, if they had smoked more than 100 cigarettes in their lifetime and whether they had smoked in the past 30 days. Those who reported both 100 cigarettes and smoking in the past 30 days were considered current smokers. Cigar and smokeless tobacco use were assessed based on use in the past 30 days. For alcohol, participants were instructed that “One drink is equivalent to a 12-ounce beer, a 5 ounce glass of wine, or a drink with one shot of liquor.” and then were asked if they had had consumed at least one alcoholic beverage in the past 30 days. To determine binge drinking, participants were asked to indicate how often they had consumed 4 or more drinks in the past 30 days. Potential alcohol abuse was assessed with the CAGE questionnaire [47]. The CAGE is so named because it asks about Cutting down drinking, being Annoyed about interventions about drinking, Guilt from drinking, and using alcohol as an Eye opener. Questions were asked about whether they (1) felt the need to cut down their drinking (2) felt annoyed by criticisms of their drinking (3) had guilty feelings about drinking and (4) had taken a morning eye opener to help them get going. Each positive response adds one point to the total score of 0–4. Scores at or above 2 are considered possible indicators of problem drinking.

### Depression

The Center for Epidemiological Studies Short Depression Scale (CES-D 10) was used to assess depression. The survey includes questions about the frequency of both feelings and behaviors during the past week and included response options of rarely or none of the time (< 1 day), some or little of the time (1–2 days), occasionally or a moderate amount of time (3–4 days), all of the time (5–7 days). The total score is the sum of points from each question with a score of four or more indicating a possible concern for depression [48].

### The perceived stress scale [PSS; [49-51]

This measure assessed how stressful participants find their lives. The ten-item measure queries how unpredictable, overloaded and uncontrollable individuals perceive their lives. In the general population, women report an average score of 16.1 (SD=7.6).

### Job satisfaction

Job satisfaction was evaluated by five questions: (1) I am optimistic about my future success with this fire department (2) I am satisfied with my job in the fire department (3) I am satisfied with the morale of the people I work with in the fire service (4) I am satisfied with my morale at the fire department (5) My work with the fire department gives me a sense of accomplishment. Each question is answered based on a 5 point Likert-type scale ranging from “very much agree” to very much disagree.”

### Approach to analysis

Descriptive statistics and rates are presented separately for career and volunteer firefighters. Published, peer reviewed data from Poston et al. [22] and Haddock et al. [24] was used for comparisons with male firefighters and these comparisons are presented in Table 1 and throughout the discussion. To determine the rates of misclassification of weight status for BMI, comparisons to both measured body fat and waist circumference are presented.

**Table 1:** Health domains of female firefighters compared to published data on male firefighters

	**Career, Female**	Career, Male*	**Volunteer, Female**	Volunteer, Male*
	**N=18**	**N=478**	**N=13**	**N=199**
***Demographics***				
Age M (SD)	33.1 yrs (8.9 yrs)	38.2 yrs (9.9 yrs)	34.2 yrs (11.1 yrs)	39.7 yrs (12 yrs)
% White	88.9%	86.4%	92.3%	97.5%
Some College or College Grad	94.4%	82.4%	92.3%	61.8%
% Married/Partnered	33.4%	72.6%	69.2%	75.4%
***Body Composition***				
Body Mass Index^Ŧ^				
Underweight	0.0%	NR	0.0%	NR
Normal	66.7%	NR	53.8%	NR
Overweight	16.7%	46.0%	30.8%	35.2%
Obese	16.7%	33.5%	15.4%	43.2%
Waist Circumference High Risk	17.6%	30.5%	38.5%	45.2%
Body Fat^¥^				
Athletic	11.1%	NR	7.7%	NR
Fitness	16.7%	NR	0.0%	NR
Acceptable	33.3%	NR	46.2%	NR
Obese	38.9%	47.7%	46.2%	54.3%
***Fitness***				
Met 12 Metabolic Equivalents (METs)	22.2%	38.7%	7.7%	23.6%
Tobacco Use **				
Current Smoker	22.2%	13.6%	15.4%	17.4%
Current Smokeless	11.1%	18.4%	0.0%	16.8%

*Data from Poston et al. 2011, NR = not reported.

**Data from Haddock et al. 2011.

Ŧ underweight (<18.5 kg/m^2^), healthy weight (range, 18.5–24.9 kg/m^2^), overweight (range, 25.0–29.9 kg/m^2^), or obese (≥30.0 kg/m^2^).

¥ athletic (14-20%), fitness (21-24%), acceptable (25-30%), obese (30% or more).

## Results and discussion

### Demographics

Similar to the estimated rates of women in the fire service from national organizations (e.g. 1, 6), 3.7% of participants (n=18) in the career department and 6.1% of participants (n=13) in the volunteer fire departments were women. Among career firefighters, the average age of participants was about 33 years and among volunteer firefighters, the average age was just over 34 years both of which were younger than the average age of males (See Table 1). Also consistent with national trends, the majority of participants identified themselves as white in both the career and volunteer departments. Among career and volunteer firefighters, most had completed some college (72.2% and 84.6% respectively) or were college graduates (22.2% and 7.7% respectively).

### General physical health

While none of the career women reported fair or poor health, only 11.1% reported an “Excellent” self-rating of their health. Based on BMI, the majority (66.7%) of career firefighters were classified in the normal weight category (range, 18.5–24.9 kg/m^2^), while 16.7% were overweight (range, 25.0–29.9 kg/m^2)^, and 16.7% were obese (≥ 30.0 kg/m^2^). Estimated body fat indicated a higher rate of obesity with 38.9% falling within the obese range and 33.3% in the acceptable range. However, when categorized using waist circumference, only 17.6% of career female firefighters were in the high risk/obese range. In general, career firefighters indicated good strength and flexibility with two thirds falling in the high range of strength and nearly the same (70.6%) falling within the good or excellent range for flexibility. Only 22.2% of career firefighters were estimated to meet or exceed the 12 MET recommendation for firefighters. Neither strength nor flexibility was significantly correlated with any of the body composition measures. Among career firefighters, 27.8% reported at least one injury in the previous 12 months. All the injuries were reported as being within the category of a dislocation, sprain or strain. More than half of participants (55.5%) believed they would have a shorter lifespan than the general population because of their position within the fire service.

Similar to female career firefighters, female volunteer firefighters were optimistic about their health with none self-reporting fair or poor general health but only 15.4% indicated their health was “excellent”. Based on BMI, approximately half (53.8%) of firefighters were classified as normal weight with the remainder being either overweight (30.8%) or obese (15.4%). Similarly, estimated body fat indicated nearly half (46.2%) of participants in the high risk/obese and the same within the acceptable range. However, 61.5% of firefighters fell within the normal range based on waist circumference. Less than 10% of volunteer firefighters met the recommended 12 MET recommendation. Similar to career firefighters, volunteer firefighters exhibited a good deal of strength with 69.2% falling within the high range of torso strength. Neither strength or flexibility was significantly correlated with body composition measures. More than half fell within the good (23.1%) or excellent (30.8%) range of flexibility. Only one participant (7.7%) reported an injury in the past 12 months among volunteer firefighters.

### Accuracy of body composition measures

Compared to measured body fat percentage, there were no false positives for obesity based on BMI, i.e., no firefighters classified as obese by BMI were found to be in the normal range based on body fat percentage. No participants were obese based on BMI but in the normal range of waist circumference. On the other hand, there were several firefighters who were classified in the non-obese range based on BMI who were found to be obese based on body fat percentage. Among career firefighters, 57.1% of women who were classified as not obese by BMI fell in the obese category by body fat standards. Similarly, among volunteers, 66.7% who were classified as non-obese by BMI were in the obese category by body fat. There were no false negatives for BMI when compared to waist circumference. Thus, BMI may underestimate obesity among female firefighters, particularly in comparison to overall body fat percentage.

### Substance use

Within the sample of female career firefighters, most had tried (83.3%) smoking cigarettes and 22.2% reported being current smokers (See Table 2). Among participants, 11.1% had tried and continue to use smokeless tobacco. Most (88.9%) of the firefighters reported drinking alcohol with the past month. Drinkers consumed alcohol on an average of 5.1 days (SD=4.0 days) in the past month and drank an average of three drinks (M=2.9 drinks, SD=1.5 drinks) on the days they drank. Most (61.1%) reported at least one binge drinking episode within the past 30 days with nearly a quarter (22.2%) reporting three or more binge drinking episodes and 11.1% reported having driven when they believed they had had too much to drink. Despite the high use of alcohol, no firefighters scored within the range of concern on the CAGE questionnaire.

**Table 2:** Health domains of career and volunteer female firefighters

	**Career, Female**	**Volunteer, Female**
	**N=18**	**N=13**
***General Health***		
Self Reported Health		
Poor	0.0%	0.0%
Fair	0.0%	0.0%
Good	44.4%	53.8%
Very Good	44.4%	30.8%
Excellent	11.1%	15.4%
Blood Pressure		
Systolic Prehypertension (120–139 mmHg)	16.7%	46.2%
Systolic Hypertension (140+ mmHg)	0.0%	7.7%
Diastolic Prehypertension (80–89 mmHg)	0.0%	0.0%
Diastolic Hypertension (90+ mmHg)	0.0%	0.0%
Strength		
Low Strength	5.9%	15.4%
Average Strength	23.5%	15.4%
High Strength	70.6%	69.2%
Flexibility		
Poor	5.6%	15.4%
Fair	5.6%	15.4%
Average	22.2%	15.4%
Good	44.4%	23.1%
Excellent	22.2%	30.8%
% Injured, past 12 months	27.8%	7.7%
***Alcohol Use***		
Drinking alcohol past 30 days	88.9%	46.2%
Average number of days drinking, past 30 M(SD)	5.0 days (4.0 days)	8.0 days (2.4 days)
Average number of drinks per day	2.9 drinks (1.5 drinks)	3.0 drinks (2.6 drinks)
% reporting binge in past 30 days	61.1%	23.1%
% reporting 3+ binges in past 30 days	22.2%	7.7%
% having driven when they had too much to drink	11.1%	0.0%
% range of concern on CAGE questions	0.0%	7.7%
***Mental Health***		
% in Range of concern, CESD*	22.2%	38.5%
Perceived Stress Scale M (SD)	12.8 (8.3)	13.5 (7.3)
Feeling nervous and stressed		
Never	5.6%	7.7%
Almost never	38.9%	23.1%
Sometimes	27.8%	38.5%
Fairly often	22.2%	7.7%
Very often	5.6%	23.1%
Feeling angered because things are outside my control		
Never	11.1%	15.4%
Almost never	33.3%	30.8%
Sometimes	33.3%	38.5%
Fairly often	16.7%	7.7%
Very often	5.6%	7.7%
Feel confident to handle problems		
Never	0.0%	0.0%
Almost never	5.6%	0.0%
Sometimes	11.1%	15.4%
Fairly often	27.8%	30.8%
Very often	55.6%	53.8%
Feeling on top of things		
Never	0.0%	0.0%
Almost never	5.6%	0.0%
Sometimes	27.8%	30.8%
Fairly often	33.3%	46.2%
Very often	33.3%	23.1%
***Job Satisfaction***		
Satisfied with job at the fire department		
Very much disagree	0.0%	0.0%
Disagree	0.0%	0.0%
Neutral	0.0%	46.2%
Agree	64.7%	15.4%
Very much agree	35.3%	38.5%
Get sense of accomplishment from work		
Very much disagree	5.9%	0.0%
Disagree	5.6%	7.7%
Neutral	11.8%	7.7%
Agree	41.2%	53.8%
Very much agree	35.3%	30.8%
Optimistic about future success with the department		
Very much disagree	0.0%	0.0%
Disagree	0.0%	15.4%
Neutral	17.6%	30.8%
Agree	52.9%	23.1%
Very much agree	29.4%	30.8%
Satisfied with personal morale at the fire department		
Very much disagree	5.9%	7.7%
Disagree	11.8%	7.7%
Neutral	29.4%	15.4%
Agree	23.5%	38.5%
Very much agree	29.4%	30.8%
Satisfied with the morale of the crew		
Very much disagree	0.0%	7.7%
Disagree	17.6%	7.7%
Neutral	29.4%	23.1%
Agree	41.2%	38.5%
Very much agree	11.8%	23.1%

*Center for Epidemiologic Studies Depression Scale.

Among female volunteer firefighters, more than half (53.8%) had tried smoking cigarettes and 15.4% reported being current smokers. Of all the firefighters, 7.7% smoked cigars and 7.7% had tried smokeless tobacco but none were current users. Rates of alcohol use were lower for volunteer firefighters than career with less than half (46.2%) reporting having consumed alcohol in the past 30 days. Of those who did drink alcohol, they drank an average of 8.0 days (SD=2.4 days) within the last 30 and they consumed an average of 3.0 drinks (SD=2.6 drinks) on the days they drank. Among the entire sample of female volunteer firefighters, less than a quarter (23.1%) reported a binge drinking episode in the 30 days prior and none reported having driven a car or vehicle after having too much to drink. Only one participant (7.7%) had a CAGE score of two or more to indicate possible problem drinking.

### Mental health

Among the career firefighters, nearly a quarter (22.2%), were within the range of concern on the CESD, suggesting they are at risk for depression. More than a quarter (27.8%) reported feeling nervous and stressed either fairly or very often and almost the same percentage reported feeling angered because things were outside of their control fairly or very often (22.3%). However, most firefighters reported strong perceived coping skills with nearly all responding that they felt confident to handle their problems and more than half reported feeling “on top of things”.

Among volunteer firefighters, more than a third (38.5%) were in the range of concern for depression based on their responses to the CESD. About a third or participants reported feeling nervous and stressed fairly or very often. In general, volunteer firefighters indicated they were confident in their abilities to manage and control the stress in their lives and most reported that they were either fairly or very often confident about their ability to handle problems.

### Job satisfaction

Career firefighters reported a high level of job satisfaction with all participants agreeing or very much agreeing that they are satisfied with their job at the fire department. Most (76.5%) agreed with the statement that their work at the fire department gives them a sense of accomplishment and nearly all (82.3%) indicated they were optimistic about their future success with their department. However, only about half (52.9%) agreed that they were satisfied with their morale at the fire department and the same percentage agreed they were satisfied with the morale of their colleagues in the fire service.

Volunteer firefighters positively endorsed items about job satisfaction for their volunteer position at a rate lower than the career firefighters with only slightly more than half stating they agreed (23.1%) or very much agreed (30.8%) with the statement that they were optimistic about their future success with the department and a similar number indicated that they agreed (15.4%) or very much agreed (38.5%) with the statement that they were satisfied with their job at the department. However, most were positive in their reports of the morale of their department with nearly a quarter 69.3% agreeing that they are satisfied with their morale and 61.6% agreeing that they were satisfied with the morale of their colleagues. Nearly all (84.6%) reported a sense of accomplishment with their work at the fire department.

## Conclusions

Overall, both career and volunteer women firefighters were optimistic about their general health and viewed their health status as either good or excellent. Body composition among firefighters and the negative health and occupational outcomes of obesity have become an important issue in the fire service and in the academic literature. Based on BMI assessment, Poston and colleagues [22] found rates of overweight/obese (BMI ≥ 25) for male career (79.5%) and volunteer (78.4%) firefighters to be higher than the general population when age-standardized. Among Non-Hispanic White women in the United States, the rate of overweight/obese is 61.2% and 33.0% for obese [52]. This study found a rate of overweight and obesity of 33.4% among career and 46.2% among volunteer female firefighters. Rates of obesity were 16.7% for career and 15.4% among volunteer female firefighters. Thus, compared both to their male colleagues and the general population, women firefighters had a more favorable body composition based on BMI.

When using other measures of body composition, women also exhibited more favorable rates of healthy body composition than their male peers. Among career firefighters, Poston et al. found 30.5% of male firefighters had a waist circumference over 40 inches; which represents increased risk of morbidity and mortality [30,53]. Among female firefighters in this study, only 17.6% had a waist circumference which would place them at health risk (waist circumference ≥ 35 inches; the cut point for females). A similar trend existed within the volunteer fire service where 45.2% of males had an increased risk based on waist circumference and only 38.5% of females were at increased risk. While rates of obesity were higher when comparing estimated body fat with BMI for both career (38.9%) and volunteer female firefighters (46.2%), the rates were lower than that found for career (47.7%) and volunteer (54.3%) male firefighters.

Available data about body composition among this subpopulation suggest several areas for future research. First, rates of obesity by any measure found rates lower among female firefighters when compared to their male colleagues. It will be important to understand whether this is a national trend or a peculiarity of the firefighters in the present study. It is possible that there are gender-related factors that influence the rates of obesity to not only be lower among female firefighters than male firefighter but also lower than general population rates for women. In addition, rates of obesity were almost double when measuring body fat compared to BMI. It has been suggested that high rates of misclassification occur when BMI is used to assess body composition due to high levels of lean muscle mass among firefighters. In the current sample, no firefighters classified as obese by BMI or waist circumference were classified as non-obese by body fat. Similar to the findings of Poston et al. [22], there was not a high rate of misclassification between obese and non-obese. Rather, when misclassification does occur, it is more common that BMI or waist circumference underestimates risk among female firefighters.

In general, career and volunteer firefighters exhibited high rates of physical fitness with regard to strength and flexibility; however, the rate of women meeting the 12 MET recommendation was lower that found among male firefighters. Research is needed to determine if these findings are consistent beyond the current sample and to identify predictors and outcomes of fitness domains among female firefighters. On limitation of the current study was the use of a non-exercise model for estimating VO_2_max. Future research should include measured VO_2_max and an analysis of the validity of non-exercise models among women firefighters who are primarily young and fit.

Tobacco use in the US fire service is a particularly interesting health behavior to examine as it has undergone a shift in rates from extremely high to now extremely low compared with the general population [24]. For career firefighters, Haddock et al. [24] found that smoking prevalence was 13.6% and 17.4% among male career and volunteer firefighters, respectively, was found to be notably lower than the general population and military service members. Findings of the current study indicate that nearly a quarter of female career firefighters and 15% of volunteers reported being current smokers. For female career firefighters, rates of use were similar to rates among women in the military (21.7%) while rates of female volunteer firefighters were lower than military samples [54]. Interestingly, rates of smoking among female career firefighters also were higher than the national average for women (17.9%) [55]. Rates of smokeless tobacco use among female career firefighters, (11.1%) was extremely high compared to the general population [56] (0.3% among women). Waldron [57] hypothesized that sex role norms and general expectations about gender appropriate behavior have led to the lower rates of female smokers in the general population. It is logical to assume that women who choose a traditionally male dominated profession also are less likely to conform to other sex role norms. Given the dangers of the occupational exposures firefighters already face fighting fighters, smoking can be particularly dangerous. Future research should examine factors which may contribute to the relatively high rates of smoking among female firefighters compared to their male colleagues and the unusually large rate of smokeless tobacco use. Also, if data turn out to be representative of the larger population of female firefighters, specific cessation interventions should be designed and targeted toward helping female firefighters quit tobacco.

Alcohol use among female career firefighters was relatively high with nearly 90% reporting consuming alcohol in the past 30 days and more than half of the firefighters reported at least one binge drinking episode in the previous month. While no published estimates of binge drinking are currently available for male firefighters, data indicates that less than a third of female military members reported a binge drinking episode in the past month. However, of note, none had scores in the range of concern on the CAGE questions indicating it is possible the drinking, while frequent, may not be resulting in negative social or occupational outcomes. Additional research should focus on understanding what accounts for the high rates of alcohol consumption. In addition, further work needs to examine the impact of the rates of alcohol use as well as the relationship between alcohol consumption and stress and coping among this population.

Nearly a quarter of firefighters were in the range of concern for depression on the CESD-10 which is higher than the national average for women of 10.1% [58]. Similar rates of concern in a screening instrument were found among women serving in the military [54]. Pyle et al. [59] found that 16% of male firefighters scored above the cut-off for range of concern on the CES-D. While the measure is to be used only for screening, the finding does indicate an area needing additional research, as firefighters have long been recognized as being at increased risk for mental health challenges due to their occupational [60-62]. Social pressures associated with working in a male-dominated profession may contribute to the elevated rates of concerning scores among this sub-group. In general, women firefighters report similar job stressors to men with the most frequent being sleep disturbance, wage/benefit concerns, safety concerns, equipment concerns, and job skills concerns (61). However, women reported experiencing significantly more occupational discrimination than their male peers (61). Particularly among career firefighters, considerably fewer were married compared to their male counterparts which also could indicate a possible area of stress. Women in the present sample reported levels of perceived stress similar to women in the general population [50-52]. Mixed findings about mental health outcomes among women suggest a need for future research on the topic.

The primary limitation of the current study is the small sample size available for analysis. While it is difficult to ensure generalizability of results given the limited number of women available for study involvement, our study was the first large-scale, population-based investigation to collect data from women in the fire service and is a solid first look at the health of women in the fire service. Despite a large sample drawn from seven states and twenty four departments, the number of available female participants for analysis was abysmally small given the nearly eight hundred male participants enrolled. In addition, the study was conducted entirely in the Midwest, so generalizability to other areas of the country is limited and findings should be considered with this limitation in mind.

In general, the findings offer an interesting glimpse of the health of women in the fire service as a generally healthy occupational workforce with some unique health risk behavior challenges. They also highlight some of the similarities and differences between male and female firefighters and bolster the argument for studying female firefighters as a unique occupational sub-population. Future research is necessary to understand in more depth the health status and health concerns of women in the fire service both as a means of maintaining the health of current firefighters and as to assist in recruitment of new firefighters.

## Abbreviations

US: United States; IAFC: International Association of Fire Chiefs; iWomen: International Association of Women in Fire & Emergency Services; IAFF: International Association of Firefighters; SCBA: Self contained breathing apparatus; LODD: Line of duty death; CVD: Cardiovascular disease; BMI: Body mass index; FEMA: Federal Emergency Management Agency; USFA: United States Fire Administration; FIRE: Firefighter Injury & Risk Evaluation Study; IRB: Institutional review board; NFPA: National Fire Protection Association; SRPA: Self Report of Physical Activity; VO_2max_: Maximal oxygen consumption; METS: Metabolic equivalent units; CESD 10: Center for Epidemiologic Studies Short Depression Scale; PSS: Perceived Stress Scale.

## Competing interests

The authors have no competing interests to report.

## Authors’ contributions

SJ participated in design of the study, data collection, data analysis/interpretation, drafted the manuscript, participated in revisions and gave final approval on the finished product. WSCP participated in design of the study, data collection, data analysis/interpretation, assisted in drafting the manuscript, participated in revisions and gave final approval on the finished product. CKH participated in design of the study, data collection, data analysis/interpretation, assisted in drafting the manuscript, participated in revisions and gave final approval on the finished product. NJ participated in design of the study, data collection, participated in revisions of the manuscript and gave final approval on the finished product. MH participated in data analysis/interpretation, participated in manuscript revisions and gave final approval on the finished product. CH participated in design of the study, participated in manuscript revisions and gave final approval on the finished product. All authors read and approved the final manuscript.

## Pre-publication history

The pre-publication history for this paper can be accessed here:

http://www.biomedcentral.com/1472-6874/12/39/prepub
